# Home-administered pre-surgical psychological intervention for knee osteoarthritis (HAPPiKNEES): study protocol for a randomised controlled trial

**DOI:** 10.1186/s13063-016-1165-z

**Published:** 2016-01-27

**Authors:** Roshan das Nair, Pippa Anderson, Simon Clarke, Paul Leighton, Nadina B. Lincoln, Jacqueline R. Mhizha-Murira, Brigitte E. Scammell, David A. Walsh

**Affiliations:** Division of Rehabilitation & Ageing, Queens Medical Centre, University of Nottingham, Nottingham, NG7 2UH UK; Department of Clinical Psychology & Neuropsychology, Nottingham University Hospitals NHS Trust, Nottingham, NG7 2UH UK; Swansea Centre for Health Economics, College of Human and Health Sciences, Swansea University, Singleton Park, Swansea, SA2 8PP UK; Arthritis Research UK Pain Centre, Clinical Sciences Building, City Hospital, Nottingham, NG5 1 PB UK; Physical Health Clinical Psychology Services, Nottinghamshire Healthcare NHS Trust, Pain Management Suite, Clinic 9, King’s Mill Hospital, Mansfield, NG17 4JL UK; School of Medicine, University of Nottingham, Room 2104, C Floor South Block, Queen’s Medical Centre, Nottingham, NG7 2UH UK; Academic Rheumatology, University of Nottingham, Clinical Sciences Building, City Hospital, Nottingham, NG5 1 PB UK

**Keywords:** Chronic pain, Cognitive behavioural therapy, Knee osteoarthritis, Depression, Anxiety, Quality of life

## Abstract

**Background:**

Knee replacement surgery reduces pain for many people with osteoarthritis (OA). However, surgical outcomes are partly dependent on patients’ moods, and those with depression or anxiety have worse outcomes. Approximately one-third of people with OA have mood problems. Cognitive behavioural therapy (CBT), a psychological therapy, is recommended by the National Institute for Health and Care Excellence for improving mood. However, evidence for the effectiveness of CBT before knee surgery in improving pain, mood, and quality of life following this surgery for people with knee OA is lacking.

**Methods/Design:**

This is a multi-centre, mixed-methods feasibility randomised controlled trial to compare treatment as usual (TAU) plus a brief CBT-based intervention with a TAU-only control, for people with knee OA. We will recruit 50 patients with knee OA, listed for knee replacement surgery, with high levels of distress (assessed using a mood questionnaire), and who consent to take part. Participants will be randomly allocated to receive TAU plus intervention or TAU. Up to 10 sessions of CBT will be offered on an individual basis by a psychologist. The assessments and interventions will be completed before surgery. Repeat assessments at 4 and 6 months after randomisation will be sent and received by post.

Two patient-partners will conduct feedback interviews with some participants to assess what aspects of the intervention were helpful or unhelpful, the acceptability of randomisation, the experience of being in a control group, and the appropriateness of the measures used. Interviews will be audio-recorded, transcribed, and analysed using the framework approach. We will examine the feasibility and acceptability of patient-partners conducting the interviews by also interviewing the patient-partners.

**Discussion:**

Findings from this study will be used to design a definitive study that will examine the clinical and cost-effectiveness of the CBT intervention in improving patient outcomes following knee surgery.

**Trial Registration:**

Current Controlled Trials 10.1186/ISRCTN80222865; Date: 19 June 2014.

## Background

Total knee arthroplasty (TKA; or joint replacement surgery) is a common elective procedure for management of chronic pain in knee osteoarthritis (OA) [1]. The success of these operations is associated with various demographic and clinical factors [2]. Predictors of good outcome are preoperative pain severity [3], body mass index, pre-operative mood, and attitudinal factors [4]. Lingard and colleagues [3] reported that preoperative pain and lower mental health scores were predictive of worse postoperative pain outcomes. Studies have also shown that preoperative depression and anxiety were associated with high pain levels 1 to 2 years after TKA [5, 6]. The prevalence of depression in men and women with OA are higher than the national average, around 33 % and 23 % respectively [7]. Data from our clinic suggested that 25 % of preoperative patients with knee OA had a Beck Depression Inventory-II score of above 13. In addition, 18 % of preoperative patients had a State Trait Anxiety Inventory-state score of above 50. These scores suggest that about a third of patients awaiting TKA experience depressed mood or increased anxiety, and they may benefit from psychological intervention.

Depression has been found to be strongly associated with preoperative pain severity [8], and the interaction between depression and chronic disease can complicate the treatment of both conditions [9]. People with OA with higher levels of optimism were more likely to have positive outcomes (including reduced pain) following surgery [4, 8]. Psychological distress may impair wound-healing [8, 10], and also has negative effects on functional outcomes and imposes role limitations in older patients a year after TKA [11]. Furthermore, concomitant depression is related to worse adherence to medical care for physical conditions [12] and greater use of pain medication [13]. The cycle of depression, fatigue, and activity avoidance has been recognised [14, 15]. In addition, comorbidity of depressive and anxiety disorders is common and has been shown to be a consistent predictor of chronicity of mood disorders [16].

Together, these studies suggest that preoperative emotional functioning is associated with postoperative pain and that a reduction in anxiety and depression pre-operatively may lead to improved post-operative outcomes.

There is a theoretical rationale, which is supported by research [4, 17, 18], for assuming that improving mood and attitudes towards surgery will result in better postoperative outcomes. For instance, studies have shown the relationship between psychological factors and wound healing, with higher anxiety and stress levels associated with greater postsurgical pain, and with pain having an effect on immune function and wound healing [17]; mood and attitudinal factors have been found to be associated with surgical outcomes, length of hospital stay, functional recovery, and patient self-report ratings of recovery [4]. However, the interventions used to address these psychological factors have been limited to ‘information giving’ preoperative classes [19–21]. None of these studies have examined the long-term impact of the intervention, nor have they examined mood-related pain and quality of life as outcomes. Furthermore, none of these studies have examined the cost-effectiveness of delivering psychological interventions in improving surgical outcomes.

Cognitive Behavioural Therapy (CBT) is a psychological treatment that has shown to be effective in treating depression and anxiety, and is considered a treatment of choice for these conditions by the National Institute for Health and Clinical Excellence [22]. TKA is a relatively costly intervention; based on the knee arthroplasty trial [23] on average, primary TKA and 5 years of subsequent care cost £7458 per patient (SD: £4058), although the benefits in terms of quality-adjusted life years (QALYs) are relatively large, with patients gaining an average of 1.33 (SD: 1.43) QALYs generating an incremental cost effectiveness ratio (ICER) of £5,623 per QALY gained [23, 24]. Improving the outcomes of TKA increases the value of the intervention to the patient and the NHS. However, there is limited research that demonstrates the clinical and cost-effectiveness of a short-term preoperative CBT-based intervention to improve postoperative TKA outcomes [25, 26]. Furthermore, there is limited research on patient-partner involvement in data collection and analysis in such studies. Therefore, evaluating this aspect within a feasibility trial may elucidate the potential challenges and benefits of such patient-partner engagement before attempting to implement this in a definitive trial.

## Methods/Design

### Trial objectives

The primary objective is to determine the feasibility of conducting the trial in line with the study protocol. The results will indicate the sample size and design of a definitive study.

Secondary objectives are to determine the following:

1. Rates of recruitment and retaining participants through the trial.
2. Acceptability of CBT for those awaiting knee surgery for OA-related pain.
3. Appropriateness of inclusion/exclusion criteria; acceptability of baseline and outcome measures, audio recording of sessions, and randomisation protocol from participants’ perspectives.
4. Sample-size needed for a fully powered Phase III randomised controlled trial (RCT).
5. The content of ‘treatment as usual’ (TAU), in order to describe this for this and future studies.
6. The content of the intervention, to inform the development of a treatment manual, by time-sampling the content of therapy.
7. The feasibility and acceptability of patient-partner led interviews and patient-partner participation in interview data analysis.
8. The feasibility of collecting data for an economic evaluation using a service-use questionnaire and understanding the main cost drivers.
9. Feasibility of conducting the interventions within existing patient pathways, that is, before TKA.

### Trial design

This is a multi-centre, mixed-methods feasibility RCT to compare TAU plus a brief CBT-based intervention with a TAU-only control with people with knee OA.

### Site and participant recruitment

Participants will be recruited from knee surgery pathways at two National Health Service (NHS) hospitals at the point when they are listed for TKA.

We will use two ways of recruiting participants: either a member of the orthopaedic clinical team will identify potential participants at the point of listing for TKA and will send out an invitation letter and participant information sheet inviting patients to complete the Hospital Anxiety and Depression Scale (HADS) and return it in a pre-paid return envelope, or the consultants and nurses will inform patients listed for TKA surgery about this study during the patients’ clinic appointments. If the patient expresses interest in finding out more, he or she will be referred to the research nurse or research associate (RA), who will explain the study, and go through the information sheet with them. The patient will be given the opportunity to ask questions about the study. The patient will be given some time alone or with the person accompanying them to consider the information, without the research nurse or RA being present. Patients will then be given the option to complete the questionnaires at that point or they will be given the reply slip, screening questionnaire, participant information sheet, and a self-addressed envelope to take away with them and post back.

Recruitment is planned to cover a 12-month period.

### Informed consent

Written informed consent will be obtained by the RA or research nurse before the participant enters the trial. Participants will have more than 24 hours before they are randomised; therefore, no treatment will begin before they have had 24 hours to consider the information. Participants will be informed that their participation is entirely voluntary, and they are free to withdraw at any time. In the event of their withdrawal, any data collected up until that point will be kept by the research team. Participants will also be asked to allow the research team access to their medical notes to obtain information on their clinical diagnosis and other medical conditions. They will be asked whether they consent to a feedback interview to assess the acceptability of the intervention and will be informed that if allocated to the intervention group, sessions may be digitally recorded to ensure treatment fidelity. The General Practitioners (GPs) and orthopaedic consultants of the consenting participants will be sent a letter to inform them of their patients’ involvement in the trial.

### Inclusion criteria

Patients will be eligible to join the trial if they i) are over the age of 18 years, ii) are listed for TKA surgery, iii) have OA of the knee, defined and scored radiologically using European League Against Rheumatism (EULAR [27]) criteria and line atlas, and iv) are reporting depression or anxiety as assessed on the HADS [28] (anxiety or depression subscale score > 7, based on Axford et al. [29]), which performs well for screening [30].

### Exclusion criteria

Potential participants will be excluded if they i) have severe co-morbid psychiatric conditions, as reported by patients or their carers (for example, dementia, or psychosis) and subsequently confirmed by checking medical notes; ii) have inflammatory arthritis (for example, rheumatoid arthritis, psoriatic arthritis, or gout); iii) are unable to provide informed consent; iv) are currently receiving psychological interventions for their mood problems (we will not exclude those on medication for their mood problems, but will record this information); and v) are unable to communicate in English, as the assessments have been standardised in English and the intervention is being delivered in English.

### Initial screening assessment

Initial screening for levels of depression and anxiety using the HADS will occur either face-to-face with the RA or research nurse or when participants return the HADS questionnaire and reply slip.

### Baseline assessment

After initial screening on the HADS, the following standardised questionnaires will be conducted to assess mood, pain, quality of life, and functional ability:

- Western Ontario and McMaster Universities Osteoarthritis Index (WOMAC) [31] to assess the pain, stiffness, and physical function.
- Intermittent and Constant Osteoarthritis Pain scale (ICOAP) [32] to assess pain.
- Beck Depression Inventory (BDI) [33] to assess the level of depression.
- Beck Anxiety Inventory (BAI) [34] to assess the level of anxiety.
- EQ-5D™ (The Euroqol Group) [35] to assess the health-related quality of life.
- Service-use questionnaire (SUQ) to assess the use of NHS and social services.

The WOMAC and ICOAP assess different aspects of pain. The ICOAP assesses intermittent and constant pain, whereas the WOMAC assesses pain, stiffness, and physical functioning. Possible score ranges for the ICOAP constant pain and intermittent pain subscales are 0 to 20 and 0 to 24, respectively, with higher scores indicating higher pain experienced. Possible ranges for the three WOMAC subscales are pain (0 to 20), stiffness (0 to 8) and physical function (0 to 68), with higher scores indicating more extreme pain, stiffness, and physical function limitations. The BDI and BAI are measures of the severity of mood disorder, and total scores range from 0 to 63 with higher scores suggesting severe depression or anxiety. The EQ-5D™ is a standardised instrument for use as a measure of health outcome. It is applicable to a wide range of health conditions and treatments; it provides a simple descriptive profile and a single index value for health status. The EQ-5D is primarily designed for self-completion by respondents and is ideally suited for use in postal surveys, in clinics, and face-to-face interviews. It is cognitively simple, taking only a few minutes to complete. Instructions to respondents are included in the questionnaire. The output from the questionnaire provides a simple descriptive profile and a single index value, which are used for health economic evaluation to enable estimation of quality adjusted life years. It is applicable to a wide range of health conditions and treatments. This study is using the five-level response category version. The ICOAP is recommended by the Osteoarthritis Research Society International, and has been Rasch analysed [36]. The Beck inventories are recommended in the Quality and Outcomes Framework guidance [37] and used extensively in OA research. These standardised assessments were selected because they have adequate psychometric properties, have been employed in other trials with this patient group, and map onto the areas our patient and public involvement (PPI) group felt were most important for people with knee OA. The SUQ is not a validated questionnaire but is based on standard practice and the resources available in the DIRUM database adapted for use in this study by health-economists, which includes the number and types of primary and secondary care NHS resources use (for example, (re)admissions, medication use, travel costs) and changes to employment status. The SUQ has been developed for the study to collect the use of NHS and social services resources by the participant during the study period.

### Randomisation

After baseline assessments, individual randomisation will be conducted by an independent party not otherwise involved with the study, using a computer-generated random code. Participants will be randomly allocated to the CBT plus TAU or TAU alone on a 1: 1 ratio.

### Sample size and justification

As this is a feasibility study, we will continue to approach and recruit people until we have randomised at least 50 participants (25 to each group). This should provide us with sufficient information to inform the design of a Phase III RCT, as the recommended sample size for feasibility trials is 12 participants per group [38].

### Intervention group

The intervention group will receive up to 10 sessions of CBT, based on general principles of CBT for anxiety and depression, and pain management, tailored to the specific needs of each participant. This is not standardised as this is a feasibility trial, so we need the flexibility to determine what is appropriate. Participants will not all require the same number of sessions because they will not all have the same severity of problems. There are no standardised protocols for CBT for osteoarthritis or pre-surgical preparation for OA, so a key part of the feasibility trial is to establish the treatment protocol. The CBT sessions will be offered by a psychologist, trained in delivering CBT-based interventions, in the participant’s home or in clinic. Some participants prefer to be seen in hospital, and some prefer to be seen at home. As this is a feasibility trial, we plan to offer the flexibility to do either so we can determine what people generally prefer. In addition, due to disability issues, some participants may struggle to make it regularly to the clinic, and by offering home sessions, we are being more inclusive. Sessions will be once or twice weekly and last approximately one hour. The content of the intervention will combine the core elements of CBT for pain management outlined by Gatchel et al. [39], Morley [40], and the Gloucester Pain Management Manual [41], namely: psychoeducation on mood and pain (for example, pain processing, the relation between pain and stress, the CBT approach to self-management, etcetera); values-based goal-setting; self-management and behavioural activation (e.g. diet, exercise, activity planning, pacing, etcetera); relaxation and mindful breathing (including instruction on the fight/flight response); cognitive restructuring (including identifying negative automatic thoughts, changing negative thinking patterns, and challenging maladaptive beliefs); and post-surgical planning (for example, acceptance of rehabilitation time-scales and adaption of coping strategies). The therapy sessions will be audio recorded. These sessions will fit within the expected waiting time for surgery (18 weeks).

### Control group (treatment as usual)

Control group participants will receive treatment as usual (TAU). They will not receive any therapeutic input from the psychologist.

All other clinical services will be provided as usual for both groups. Any additional input (including medical or psychological interventions) participants receive during the study will be recorded on the SUQ.

### Compliance with interventions

The psychologist will record the number of treatment sessions participants receive. To ensure the fidelity of the intervention, the content of the treatment sessions will be digitally recorded, transcribed and analysed. Time-sampling, based on minute-by-minute coding of content, and saliency analysis of the intervention transcripts will enable us to document the content and delivery of the intervention. This will enable us to further develop the manual for the Phase III RCT.

### Participant outcome measures

Participants from both groups will be assessed 4 and 6 months after randomisation, using the same assessments as at baseline. Paper-based questionnaires including the outcome measures will be sent to participants by post, with a pre-paid return envelope. If participants have difficulty completing the measures, they will be able to request help by telephone from the RA. As far as is feasible, medication prescribed will be checked in the patients’ hospital notes and compared to findings from the SUQ and any critical discrepancies will be noted. This exercise will inform the development of the SUQ for the main study.

### Minimisation of bias

The participants will not be blind to the allocated intervention. Outcome assessments will include self-report questionnaires, which will be sent to participants by post and will be received by the RA who will remain blind to group allocation. To prevent unblinding, the RA will request participants not to discuss any aspect of being involved with the study. The RA will also be required to guess the treatment allocation for each participant (that is, whether they are ‘intervention’ or ‘TAU’), and this will be compared later to the actual allocation to determine the degree of unblinding. As participants will know to which group they were allocated, observer-outcome blinding will be possible.

### Participant feedback interviews

Up to 30 participants (15 from the intervention and 15 from the TAU groups) will be invited to take part in a brief semi-structured feedback interview conducted by the RA. Selection will be purposive to capture data from both ‘typical’ participants (complete the intervention, no complications, etcetera) but also from those who experience difficulties with therapy or research process, complications and/or drop out of the study. Thirty is a manageable number to interview in the context of a study of this size, and a total population of 30 should provide sufficient data to be confident in the study findings. Moreover, prior research has identified that data saturation occurs after approximately 12 interviews [42, 43], which means that we might consider intervention and TAU groups as distinct data sets if we wish to draw conclusions about one side of the study or other. Up to 10 of these interviews will be conducted by trained and supported patient-partners, who will be requested to keep a research diary of their experiences, including the challenges of conducting these interviews. We believe this patient-partner activity is appropriate because some participants may feel more comfortable talking freely about their experiences of the study/intervention with someone who has prior experience of the same health condition, and because this person will have greater independence from the academic research team. We will provide training and support for patient-partners and allocate funding to cover these costs.

These interviews will offer intervention participants an opportunity to report on what they found useful or unhelpful about the intervention, the content, and delivery (including format and ‘dose’ of treatment). Control group participants’ interviews will explore their feelings about not receiving the intervention and to ascertain the acceptability of randomisation. For both groups we will make inquiries about the services they received to add to and contextualise the information collected from the SUQ. This will enable us to describe the TAU. All interviewed participants will be asked about the research process, focussing on recruitment, randomisation, and outcome measures.

### Duration of participant participation

**Fig. 1:**
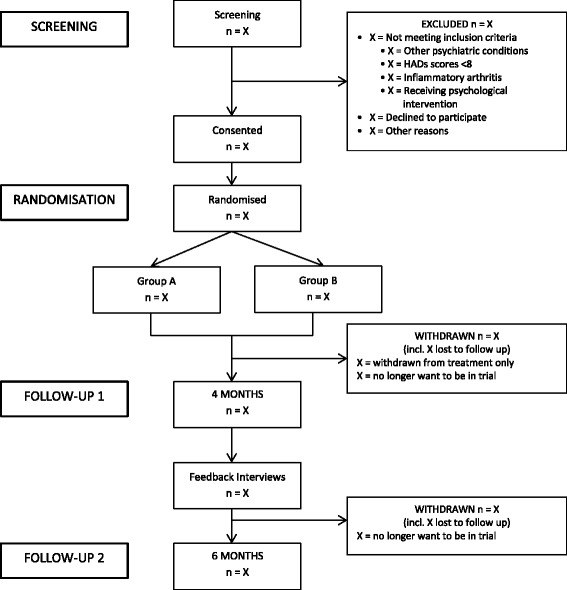
CONSORT diagram. The CONSORT diagram illustrates and reports the progression of the participants through the different points of the study

Figure 1 shows the expected progress of the study. Each participant is expected to be in the study for approximately 8 months, including 6-month follow-up questionnaires and qualitative interviews. Participants will leave the study when they have completed the 6-month follow-up. See the Trial CONSORT Diagram in Fig. 1.

### Data analysis

The outcomes map onto the aims of the feasibility study (see Table 1) and will be assessed in the following three ways:

**Table 1:** Trial objectives, outcome measures and sources of data. For each trial objective, the outcome measure to be reported and the sources of the data are described. Trial source data include screening and recruitment logs, CONSORT diagram, and case report forms at the different time points (baseline, 4 months follow-up (1) and 6 months follow-up (2))

Objectives	Outcome measures	Data source
1. Rates of recruitment and retaining participants through the trial	The number of patients listed for TKA surgery	Screening logs
		Study CONSORT diagram (screening)
	Number of patients recruited (giving consent) and	Recruitment logs
		Study CONSORT diagram (randomisation)
	Number completing follow-up 1	Recruitment logs
		Study CONSORT diagram (follow-up 1)
	Number completing follow-up 2	Recruitment logs
		Study CONSORT diagram (follow-up 2)
	Number withdrawing from the trial	Recruitment logs
		Study CONSORT diagram (randomisation, follow-up 1 and 2)
2. Acceptability of CBT for those awaiting knee surgery for OA-related pain	Number of participants receiving the intervention	Treatment records
		Study CONSORT diagram (randomisation - received allocated intervention)
	Number of sessions completed	Treatment records
	Number of participants who did not receive allocated treatment (that is, did not start treatment and discontinued treatment)	Treatment records
		Study CONSORT diagram (randomisation - did not receive allocated intervention; follow-up - discontinued intervention)
	Participant views of treatment	Participant feedback interviews.
3a. Appropriateness of inclusion/exclusion criteria	Scores and rates of mood problems at screening	Screening questionnaires
		Study CONSORT diagram - number not meeting study inclusion criteria
		Participants’ medical notes
3b. Acceptability of baseline and outcome measures	Rates of completion of questionnaires	Study CONSORT diagram – Follow-up (number of questionnaires returned and number lost to follow-up at 4 and 6 months after randomisation)
	Amount of missing items from questionnaires and whether this data was obtained with telephone follow-up	Outcome questionnaires
	Specific items consistently missed from questionnaires	Outcome questionnaires
	Ease of answering outcome questionnaires	Participant feedback interviews
3c. Audio recording of sessions	Number of participants consenting to sessions being audio recorded	Consent forms
	Number of participants consenting to be randomised	Study CONSORT diagram (randomisation)
3d. Randomisation protocol from participants’ perspectives	Participants’ views of the randomisation protocol	Participant feedback interviews
4. Sample-size needed for a fully powered Phase III randomised controlled trial (RCT)	Power and sample size calculations based on descriptive statistics	Quantitative data: baseline and outcome measures
5. The content of ‘treatment as usual’ (TAU), in order to describe this for this and future studies	TAU for SFH and NUH	Participant feedback interviews
		Data from the service use questionnaire (SUQ)
6. The content of the intervention to inform the development of a treatment manual by time-sampling the content of therapy	Time-sampling (minute by minute coding of content)	Treatment audio recordings
	Saliency of analysis of intervention transcripts	Treatment audio recordings
		Participant feedback interview transcripts
7. The feasibility and acceptability of patient-partner led interviews, and patient-partner participation in interview data analysis	Number of participants consenting to be interviewed by a patient-partner	Study monitoring database
	Perceived challenges of conducting interviews	Patient-partner research diaries
	Views on the effectiveness of this participatory research model	In-depth interviews with patient partners
	Quality and consistency of interview data	Interview transcripts
8. The feasibility of collecting data for an economic evaluation using a service-use questionnaire and understanding the main cost drivers	Rates of completion	Service-use questionnaire
	Participant views of questionnaire items	Participant feedback interviews
9. Feasibility of conducting the interventions within existing patient pathways, that is, before TKA	Number of participants who do not complete treatment before their surgery date	Treatment attendance records
		Study CONSORT diagram

1. Individual feedback interviews with study participants
  The interview data generated will be subject to a framework analysis (see below). We will also compare the content of interviews conducted by patient-partners and the RA. Two researchers (RdN and PL) will review anonymised interview transcripts to ensure the quality and consistency of the interview data across the sample.
2. Completion of outcome assessments and evaluation of intervention content and delivery
  Rates of meeting eligibility criteria, recruitment, consent, completing treatments, and completing outcome questionnaires will be described/reported in the CONSORT diagram. We will also record what data was found to be missing from the outcome questionnaires and whether this missing information was obtained with telephone follow-up. This method of reducing missing data has been found useful in other studies [44]. These data (and information from participant interviews) will also indicate any problems with outcome questionnaires being sent by post. The scores from the questionnaires will enable us to compute sample-size and power calculations for the future Phase III RCT. The feasibility of using the SUQ will also be assessed by examining rates of completion. By recording the dates of recruitment, randomisation, surgery, and keeping treatment attendance records, we will be able to assess how the intervention fits within the existing patient pathway.
  Time-sampling, based on minute-by-minute coding of content, and saliency analysis of intervention recordings will enable us to document the content and delivery of the intervention. This will help us develop the final manual for the Phase III RCT.
3. In-depth individual interview and research diary evaluation with the patient-partners
  The effectiveness of this participatory research model will be evaluated through an examination of the research diaries, and through an in-depth interview with patient-partners (conducted by RdN or PL). These interviews will also be audio recorded and transcribed verbatim.

#### Qualitative analyses

Qualitative data will be analysed using a framework approach [45–47]. This is a hierarchical, matrix-based method developed for applied qualitative research. It is particularly suited to research where the goals are clearly defined at the onset, for example, to support the development of a future trial. An initial thematic framework will be constructed from the interview objectives and existing literature (for example, literature on clinical trial recruitment and randomisation in research). The interview data will then be mapped onto this framework, and the framework will be amended if required to include new concepts introduced during the interviews. After all the data have been ‘charted’ in this manner, tables/matrices will be used to summarise each main theme and interpreted to address the interview objectives. Study participant interviews will be analysed in conjunction with the patient-partners. The draft thematic framework for the participant interviews will be fed back to the PPI steering group for their comments. We will incorporate their comments in the final analysis of the thematic framework. Elements of the thematic framework identified will be substantiated with anonymised participant quotes.

#### Quantitative analysis

For the quantitative data, we will use descriptive statistics (using the statistical software package SPSS version 21) to describe the outcomes, and to inform power and sample size calculations for a future definitive study. Based on the characteristics of the data, appropriate parametric or non-parametric statistics will be used. All tests will be two-sided with an alpha level of 0.05.

### Health economics evaluation

Descriptive analyses will be undertaken of both the EQ-5D™ responses (scored using the algorithm provided by the developers and the visual analogue scale scores) and SUQ data. Estimates of the cost of providing the intervention, the main cost drivers in terms of health care resource use, the impact of receiving the intervention prior to TKA on EQ-5D™ and quality adjusted life years will be presented. We will make estimates of the cost effectiveness of the intervention expressed as an incremental cost effectiveness ratio together with exploration of the impact of uncertainty. These analyses will be important for informing the main study data collection and the methodological challenges for undertaking a full economic evaluation.

### Assessment of safety and adverse events

The occurrence of adverse events as a result of participation within this study is not expected, since the trial involves the use of an evidence-based psychological intervention delivered by a suitably qualified psychologist. Therefore, no adverse event data will be collected. If a patient is identified as suicidal, usual clinical procedures will be followed by the clinical team responsible for the patient’s care.

If a potential participant is identified as having a high score on the HADS (>15) at baseline, we will recommend that they contact their GP to discuss this further. If a participant scores high on Question 9 on the BDI (‘I would kill myself if I had the chance’), a risk assessment will be undertaken, and we will advise them to discuss this score with their GP or contact the appropriate crisis team. This will not affect their participation in the study. If, however, they have been offered psychological therapy by routine NHS clinical services at the time of assessing eligibility, they will not be eligible for inclusion. In practice, this is unlikely, given the long waiting times for people to receive such therapies. If they are unable to participate in the trial treatment, this will indicate the upper limit of distress which may need to be applied as an exclusion criterion in future studies. In the unlikely event that a participant is found to have a suicidal risk, the recruiting NHS Trust’s standard suicide risk protocol will apply.

The patient-partners will have full UK Disclosure and Barring Service (DBS) clearance. Home visits will be conducted in accordance with our Trust’s lone working policy.

### Participants who withdraw

Participants will be informed that they are free to withdraw from the study at any time without it affecting their usual health care or legal rights. Participants will be withdrawn from the study if consent is withdrawn. However, they will be made aware that data collected before their withdrawal cannot be destroyed and may still be used in analysis but no identification will be possible. If a participant indicates a wish to withdraw from the intervention, we will enquire whether they will consider completing the primary outcomes. Participants who withdraw from the study will not be replaced, but withdrawal rates and reasons for withdrawal (where provided) will be recorded. Some participants may be unable to complete all therapy sessions. This information will be recorded.

Withdrawal from this therapy is not considered a safety issue for participants.

### Criteria for terminating the study

The trial will not be terminated unless instructed by the funder, sponsor, or ethics committee, but failure to comply with treatment will be recorded as an outcome.

### Trial management

A Trial Management Group (TMG) (TMG: das Nair, Lincoln, Scammell, Walsh, Clarke, Anderson and Mhizha-Murira) will meet fortnightly in the first 2 months and thereafter monthly to ensure that milestones are achieved. The progress of the study will be monitored by the Arthritis Research UK Pain Centre management group (who meet every 4 months), which is chaired by Walsh and includes other members of the Pain Centre staff with clinical expertise in arthritis and pain. They have agreed to act as an advisory group. Finally, the progress of the study will also be reported to the PPI Advisory Panel at four meetings over the course of the study through one of our patient-partners on this study.

The RA will be project manager and will be supervised by the Chief Investigator (CI). The CI has overall responsibility for the study and shall oversee all study management.

### Definition of a protocol deviation

A protocol deviation is an unintentional or unanticipated departure from the expected conduct of a study that is inconsistent with the protocol, consent documents, or other study procedures. All protocol deviations will be recorded by the PIs, and the CI will be notified.

### Ethical approval

Ethical approval was obtained from the NRES Committee East Midlands – Nottingham 1 on 20.03.2014 (ref: 14/EM/0099).

## Discussion

This study was conceptualised in response to a themed call for ‘surgical research’ from the National Institute for Health Research in 2012 and in response to the expressed need from our patients.

We anticipate that one of the biggest challenges to recruit to this study will be the precise timing of surgery in relation to when patients are first listed for TKA. Because we have a limited window in which to complete the therapy sessions, a considerable amount of flexibility would be required from both the participants and the therapist. They will need to work flexibly throughout the week, in order to cater to participants’ availability. Furthermore, as TKA is an ‘elective’ surgical procedure, some patients may have or may choose to have their surgery postponed. If this happens after randomisation, there may be issues in delivering the intervention and collecting follow-up data, particularly if this coincides with the participant having the surgery (and associated pain or discomfort) at that time. However, as a feasibility trial, these are some of the issues that we hope to understand better to inform the design of a Phase III RCT.

## Trial status

The first site was open to recruitment on 4 July 2014, and the first participant consented on 21 July 2014. The second site was open to recruitment on 7 August 2014 and the first participant there was consented on 1 October 2014. At the time of preparing this manuscript, 41 people have been consented and randomised. Recruitment is due to finish at the end of June 2015.

## Abbreviations

CBT: cognitive behaviour therapy
CI: chief investigator
GP: general practitioner
HADS: hospital anxiety and depression scale
NHS: National Health Service
OA: osteoarthritis
PI: principal investigator at a local centre
PPI: patient-public involvement
RA: research associate
RCT: randomised controlled trial
SUQ: service use questionnaire
TAU: treatment as usual
TKA: total knee arthroplasty
TMG: Trial Management Group
